# Rapid screening and identification of bioactive compounds specifically binding to beta 2-adrenoceptor from San-ao decoction using affinity magnetic fine particles coupled with high-performance liquid chromatography–mass spectrometry

**DOI:** 10.1186/s13020-018-0207-8

**Published:** 2018-09-24

**Authors:** Fuhuan Fei, Huanmei Sun, Xixi Cheng, Jiajun Liu, Jing Wang, Qian Li, Yajun Zhang

**Affiliations:** Key Laboratory of Resource Biology and Biotechnology in Western China, Ministry of Education, College of Life Sciences, Northwest University, Xi’an, 710069 China

**Keywords:** Affinity selection methods, Beta 2-adrenoceptor, Bioactive compounds, Magnetic fine particles, San-ao decoction

## Abstract

**Background:**

San-ao decoction (SAD) has been widely used in Chinese medicine against respiratory diseases, such as asthma and rhinallergosis. The bioactive compounds for such pharmacological action remain unknown.

**Methods:**

We developed a methodology to isolate the bioactive compounds of SAD. The assay involved the immobilization of beta 2-adrenoceptor (*β*_2_-AR) onto magnetic fine particles, the capture of target compounds by the immobilized receptor, the identification of the receptor bound compounds by reversed-phase high-performance liquid chromatography coupled with tandem mass spectrometry.

**Results:**

Vicenin, shaftoside, isoshaftoside, liquiritin apioside and isoliquiritin apioside were identified as *β*_2_-AR ligands in SAD extract. The binding of these compounds to *β*_2_-AR occurred on serine^169^, serine^170^ and phenylalanine^256^ of the receptor.

**Conclusions:**

The developed methodology has high stability and specificity for recognizing and isolating target compounds. It is an alternative method for rapidly screening bioactive compounds of immobilized receptor from Chinese prescriptions.

**Electronic supplementary material:**

The online version of this article (10.1186/s13020-018-0207-8) contains supplementary material, which is available to authorized users.

## Background

Drug discovery involves identifying compounds that prevent or control diseases and play an important role in maintaining human health [1–3]. Despite increased investments in manpower, materials and financial resources for pharmaceutical research, the number of innovative drugs approved has declined during the last few decades [4, 5]. This is mainly due to the incompatibility between the increasing difficulties in drug discovery and the relatively low efficiency of the current lead screening methods. Efforts to create new, more efficient methodologies are urgently needed.

Natural products are the main source of virtually all medicinal preparations, and between 1981 and 2010, 34% of approved small molecule drugs were from natural products [6]. The impressive contribution of natural products to medicine, including traditional Chinese medicine, has promoted their use in driving drug discovery [7–9]. The classic approaches depend on the bioassay-guided screening of natural products, such as animal- and cell-based methods, have been continuously improved to utilize technological advances and achieve high throughput [10]. More recently, developed functional assays and phenotypic screens, such as cellular membrane affinity chromatography and computer-aided virtual screening technologies have received increasing attention [11–15]. In our previous work, beta 2-adrenoceptor (*β*_2_-AR) chromatography was established for the identification of bioactive compounds of the receptor [16]. This strategy has limitations of time-intensity and labor-intensity in preparation of chromatographic columns. The structure of *β*_2_-AR may misfold during the high pressure environment. Here, we established a bio-affinity technique by the utilization of magnetic material to remove the isolation and identification bottlenecks in identifying procedures [17–21]. Magnetic fine particle (MFP)-based ligand isolation methods are highly desirable because they utilize the unique properties of MFPs, such as convenient solid–liquid separation, high surface area and good biocompatibility [22–24]. Subsequently, with the molecular docking technology, we predicted the potential activity of bioactive compounds obtained from ligand capture [25].

San-ao decoction (SAD) is a basic prescription for treating respiratory diseases in Chinese practice. The formula is indexed in the Pharmacopoeia of the People’s Republic of China 2015, and consists of three herbs: *Herba Ephedrae*, *Semen Armeniacae Amarum* and *Radix Glycyrrhizae*. Pharmacologically, respiratory ailments are mainly mediated by the *β*_2_-AR, which belongs to the G-protein coupled receptor (GPCRs) superfamily [26, 27]. We hypothesized that SAD contains bioactive compounds that bind to *β*_2_-AR. In this work, we immobilized *β*_2_-AR onto MFPs to construct a new biologically relevant isolation material. The immobilized *β*_2_-AR MFPs were used to rapidly screen bioactive compounds of the receptor from SAD. The compounds of interest were subsequently isolated and identified by high-performance liquid chromatography–tandem mass spectrometry (HPLC–MS/MS). Molecular docking confirmed the binding between the bioactive compounds and *β*_2_-AR. The receptor-functionalized MFPs provide a convenient and effective strategy for isolating ligands from traditional Chinese medicines.

## Methods

The Minimum Standards of Reporting Checklist contains details of the experimental design, and statistics, and resources used in this study (Additional file 1).

### Chemicals and instruments

Ferric chloride hexahydrate (FeCl_3_·6H_2_O) (99%) and ferrous chloride tetrahydrate (FeCl_2_·4H_2_O) (99%) were purchased from Tianjin Tianli Chemical Reagents Ltd (Tianjin, China). Analytical-grade sodium hydroxide, trisodium citrate, ethanol and aqueous ammonia (25 wt%) were acquired from Shanghai Chem. Reagent Co. (shanghai, China). Reference standards of salbutamol (batch no. 100328–200703), terbutaline (batch no. 100273–201202) and shaftoside (batch no. 111912–201703) were purchased from the National Institutes for Food and Drug Control (Beijing, China). Vicenin (batch no. B21399), isoshaftoside (batch no. B21563), liquiritin apioside (batch no. B25831) and isoliquiritin apioside (batch no. B20987) were purchased from Shanghai yuanye Bio-Technology Co., Ltd (Shanghai, China). *Herba Ephedra, Semen Armeniacae Amarum* and *Radix Glycyrrhizae* were obtained at a local medicinal market and identified by Professor Xiao Chaoni in the College of Life Sciences of Northwest University.

An AKTA10 low-pressure chromatographic system from GE Healthcare Life Sciences (Uppsala, Sweden) was utilized for *β*_2_-AR purification. Chromatographic analysis of the drugs were performed on an EClassical 3100 series apparatus (Dalian Elite Analytical Instruments Company, Dalian, China) equipped with an isocratic pump, a column oven and an ultraviolet–visible detector. Separation and identification of the bioactive compounds were carried out on an Agilent 1100 series of high-performance liquid chromatography (Santa Clara, CA, USA) coupled with an SL trap mass spectrometer (Waldbronn, Germany). The size distribution of MFPs was determined by dynamic light scattering (DLS) using a Malvern ZS ZEN 3600 system (Malvern, Worcestershire, UK).

### Preparation of SAD

SAD was extracted by heat reflux [28]. Briefly, nine grams of each herb (*Herba Ephedrae*, *Semen Armeniacae Amarum* and *Radix Glycyrrhizae*) were weighed according to the Pharmacopoeia formula dosages. The mixture was decocted three times with 8 volumes of water (v/w) and 1.0 h for each time. The three filtrates were combined and concentrated to a certain volume via evaporation. Appropriate volumes of ethanol were added to the concentrated solution to give a final ethanol concentration of 80%. The mixture was stored for 12.0 h under an ambient atmosphere and was collected by filtration. The filtrate was concentrated to 1.0 g/mL under vacuum at 40 °C and stored at 4 °C for further experiments.

### Synthesis of magnetic fine particles

Magnetic fine particles were synthesized by chemical coprecipitation based on the reaction of Fe^3+^ + Fe^2+^ + OH^−^ → Fe_3_O_4_ [29]. We dissolved FeCl_3_·6H_2_O (10.812 g, 40 mM) and FeCl_2_·4H_2_O (3.9742 g, 20 mM) in 150 mL of distilled water. Oxygen was removed by a nitrogen stream for 1.0 h. An aliquot of 20 mL aqueous NaOH solution (8.0 g, 10 mM) was added to the mixture slowly and uniformly using a drop funnel. The solution was mechanically stirred for 1.0 h until the color turned black. The black solution was heated to 90 °C and then maintained at 90 °C for 1.0 h to ripen the particles. Decreasing the temperature to 25 °C, we collected the magnetic material using a magnet. The collected material was dispersed in 200 mL of 0.3 M sodium citrate solution and heated for 1.0 h at 80 °C. The solvent was removed after collecting the magnetic Fe_3_O_4_ FPs using a magnet. The Fe_3_O_4_ FPs were thoroughly rinsed with acetone and kept at 30 °C until all the solvent was removed. The dried Fe_3_O_4_ FPs were modified with aminopropyltriethoxysilane (APTES) to synthesize Fe_3_O_4_@NH_2_ FPs [30].

### Preparation and purification of *β*_2_-AR

**Fig. 1:**
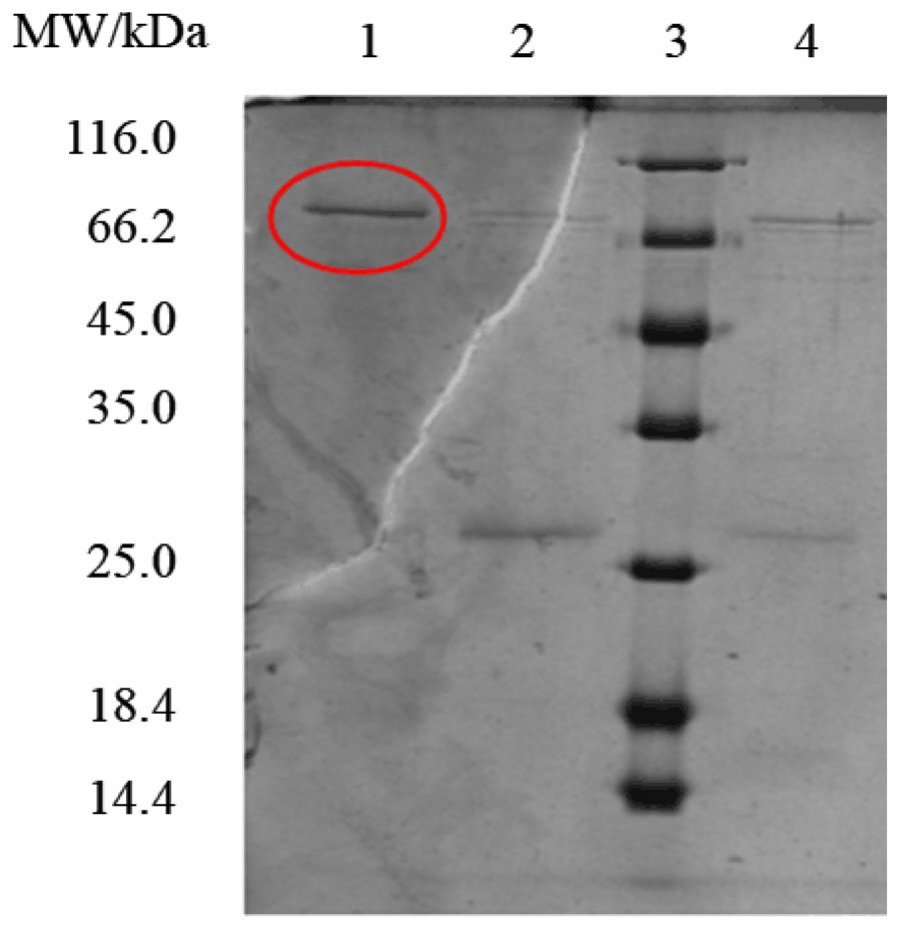
SDS-PAGE analysis of *β*_2_-AR. Lane 1, collection from Quaternary Sepharose Fast Flow column gradiently eluted by 18–26% phosphate buffer; Lane 2, collection from Quaternary Sepharose Fast Flow column gradiently eluted by 26–40% phosphate buffer; Lane 3, protein molecular weight marker (the molecular weights from top to bottom were 116.0, 66.2, 45.0, 35.0, 25.0, 18.4 and 14.4 kDa, respectively); Lane 4, collection from Quaternary Sepharose Fast Flow column gradiently eluted by 40–50% phosphate buffer. The concentration of phosphate buffer was 20 mM with the presence of 0.8 M NaCl

His-tagged *β*_2_-AR was prepared and purified according to the methods in our previous work [31]. Briefly, genetically engineered bacteria (*E. coli* BL21 (DE3)-pET32a-*β*_2_-AR) were incubated in 50 mL of Luria–Bertani medium containing 100 μg/mL penicillin. When the OD value reached 0.4–0.6, isopropyl *β*-d-thiogalactopyranoside was added to the medium at a final concentration of 2.0 mM to induce the expression of the receptor. Ni-chelated Sepharose high-performance affinity column and Quaternary Sepharose Fast Flow column were utilized to purify *β*_2_-AR by sequential. The fraction of interest was collected by eluting the Ni-chelated Sepharose high-performance affinity column using 50% phosphate buffer (20 mM, containing 0.5 M NaCl and 0.5 M imidazole, pH 7.4). The purification of the collected fraction was performed on the Quaternary Sepharose Fast Flow column. The elution containing *β*_2_-AR was collected from the column by 20 mM phosphate buffer (containing 0.8 M NaCl, pH 7.4) with gradient elution ranging from 18 to 50%. The purity of *β*_2_-AR was measured by sodium dodecyl sulfate-polyacrylamide gel electrophoresis (SDS-PAGE). The fraction with greater than 90% purity was used for further investigation (Fig. 1).

### Immobilization of *β*_2_-AR

**Fig. 2:**
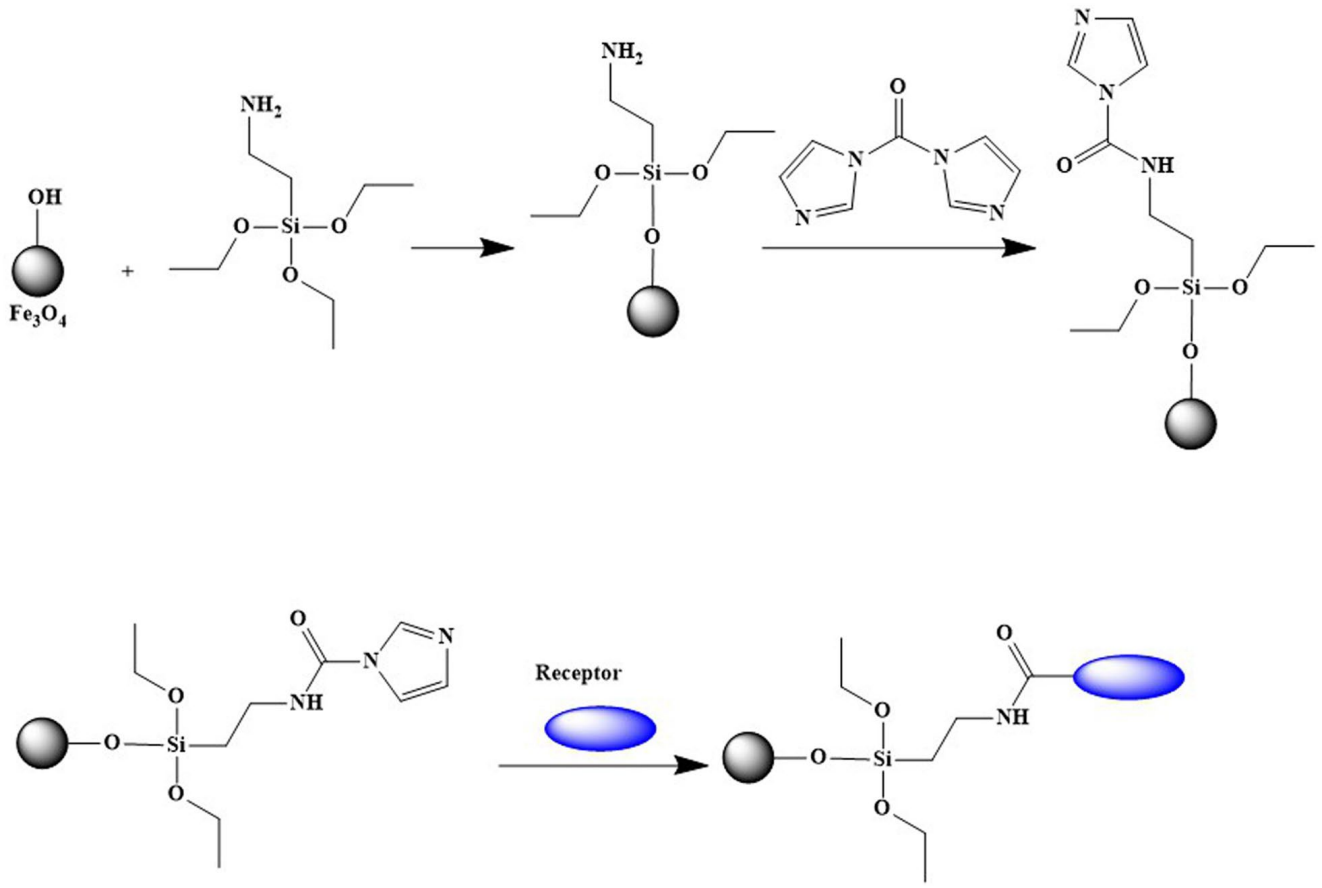
The scheme for the immobilization of *β*_2_-AR on the surface of Fe_3_O_4_ FPs. The structure of the compound was created using Chemdraw software. The blue ellipsoid represented *β*_2_-AR

As illustrated in Fig. 2, the purified *β*_2_-AR was immobilized on the surface of Fe_3_O_4_@NH_2_ FPs. In this case, we suspended 0.5 g of Fe_3_O_4_@NH_2_ FPs in 50 mL acetonitrile containing 0.5 g N,N′-carbonyldiimidazole. The mixture was stirred for 6.0 h to activate the fine particles. After the removal of acetonitrile, the activated Fe_3_O_4_@NH_2_ FPs were resuspended in 20 mM phosphate buffer and mixed with 10 mL of 0.24 mg/mL purified *β*_2_-AR. The solution was mechanically stirred for 3.0 h at 4 °C to accomplish the reaction between the receptor and the imidazole residue on the Fe_3_O_4_@NH_2_ FPs. The modified Fe_3_O_4_@*β*_2_-AR FPs were collected using a magnet, while the unbound *β*_2_-AR was removed. The obtained Fe_3_O_4_@*β*_2_-AR FPs were suspended in 1% glycine solution and stirred for 30 min to quench the unreacted imidazole groups. The final Fe_3_O_4_@*β*_2_-AR FPs were collected and washed three times with phosphate buffer. DLS was used to determine the size distribution of the Fe_3_O_4_@*β*_2_-AR FPs.

### Bioactivity of the immobilized *β*_2_-AR

Salbutamol and terbutaline are both specific agonists of *β*_2_-AR. They have similar structures such as meta-hydroxyl group, phenyl ring and N-terminus. Because of the same binding sites, salbutamol can be competitively displaced by terbutaline without any influence on the protein structure. Using the selective *β*_2_-AR agonists salbutamol and terbutaline as probes, the bioactivity of Fe_3_O_4_@*β*_2_-AR FPs was investigated by displacement experiments. Fifty milligrams of Fe_3_O_4_@*β*_2_-AR FPs were incubated in 200 μL of salbutamol (10 μg/mL) and gently shaken for 30 min. The unbound salbutamol was discarded after collecting the fine particles using a magnet. The result Fe_3_O_4_@*β*_2_-AR/salbutamol FPs were rinsed twice with 1.0 mL ammonium acetate buffer (10 mM, pH 7.4) and incubated with 100 μL competitive agent (terbutaline, 2.0 μg/mL) for 30 min to release the bound salbutamol [32]. The supernatant was collected after the fine particles were sequestered by a magnet. A similar procedure was performed with a control group of Fe_3_O_4_@NH_2_ FPs, The drug concentration in the collected solution was determined by HPLC using an Agilent C_8_ column (5 μm, 4.6 × 150 mm) with a mobile phase of 10% (v/v) methanol/water containing 0.1% (v/v) formic acid, a flow rate of 0.6 mL/min and a detection wavelength of 276 nm.

### Screening of bioactive compounds in SAD

An aliquot of 50 mg Fe_3_O_4_@*β*_2_-AR FPs was suspended in 200 μL of 0.2 g/mL SAD extract. This suspension was gently shaken for 30 min at room temperature. Following the removal of solvent, we washed the fine particles twice using 1.0 mL of 10 mM ammonium acetate. Bioactive compounds bound to the immobilized receptor were collected by treating the cleaned fine particles with 100 μL terbutaline (0.01 mg/mL) as a competitor. Terbutaline, a strong *β*_2_-AR binder, has capacity to target all the three types of binding sites of *β*_2_-AR [33]. As a result of competitive interaction, terbutaline can fully displace the potential ligands from the receptor. Simultaneously, this strategy is capable of probing the exact binding sites of the bound ligands inspired by the competitive displacement.

The released bioactive compounds were further separated and identified by HPLC–MS/MS with electrospray ionization (HPLC–ESI–MS/MS). An Agilent TC-C_18_ column (5 μm, 4.6 × 250 mm) was used with 40% (v/v) methanol/0.1% (v/v) formic acid as mobile phase. The flow rate was 0.6 mL/min and the column temperature was 30 °C. The nebulizing gas pressure was set at 50 psi. The flow rate and temperature of the dry gas were 10.0 L/min and 350 °C, respectively. Mass spectra were acquired in negative mode with scan range of 50–1000 amu.

### Molecular docking

The crystal structure of *β*_2_-AR was constructed by homologous modeling according to a previous work that utilized the methylated *β*_2_-AR–Fab complex (PDB ID: 3KJ6) as the template. The structures of vicenin, shaftoside, isoshaftoside, liquiritin apioside and isoliquiritin apioside were created by ChemDraw Ultra 8.0 software (PerkinElmer Inc., Waltham, MA, USA), and was converted to pdbqt format using Chem3D Ultra 8.0 software (PerkinElmer Inc., Waltham, MA, USA) followed by MM2 energy minimization. Diverse receptor–ligand complexations were generated by AutoDock 4.2 which was downloaded from the Scripps Research Institute website (La Jolla, CA, USA). A grid box size of 62 × 56 × 50 points in the x, y and z directions was built to locate the receptor ligand binding domain. The grid center was set at x = 0.644, y = − 2.121 and z = − 7.094, which was large enough to accommodate the three known types of binding sites in *β*_2_-AR.

## Results

### Morphological characterization of immobilized *β*_2_-AR

**Fig. 3:**
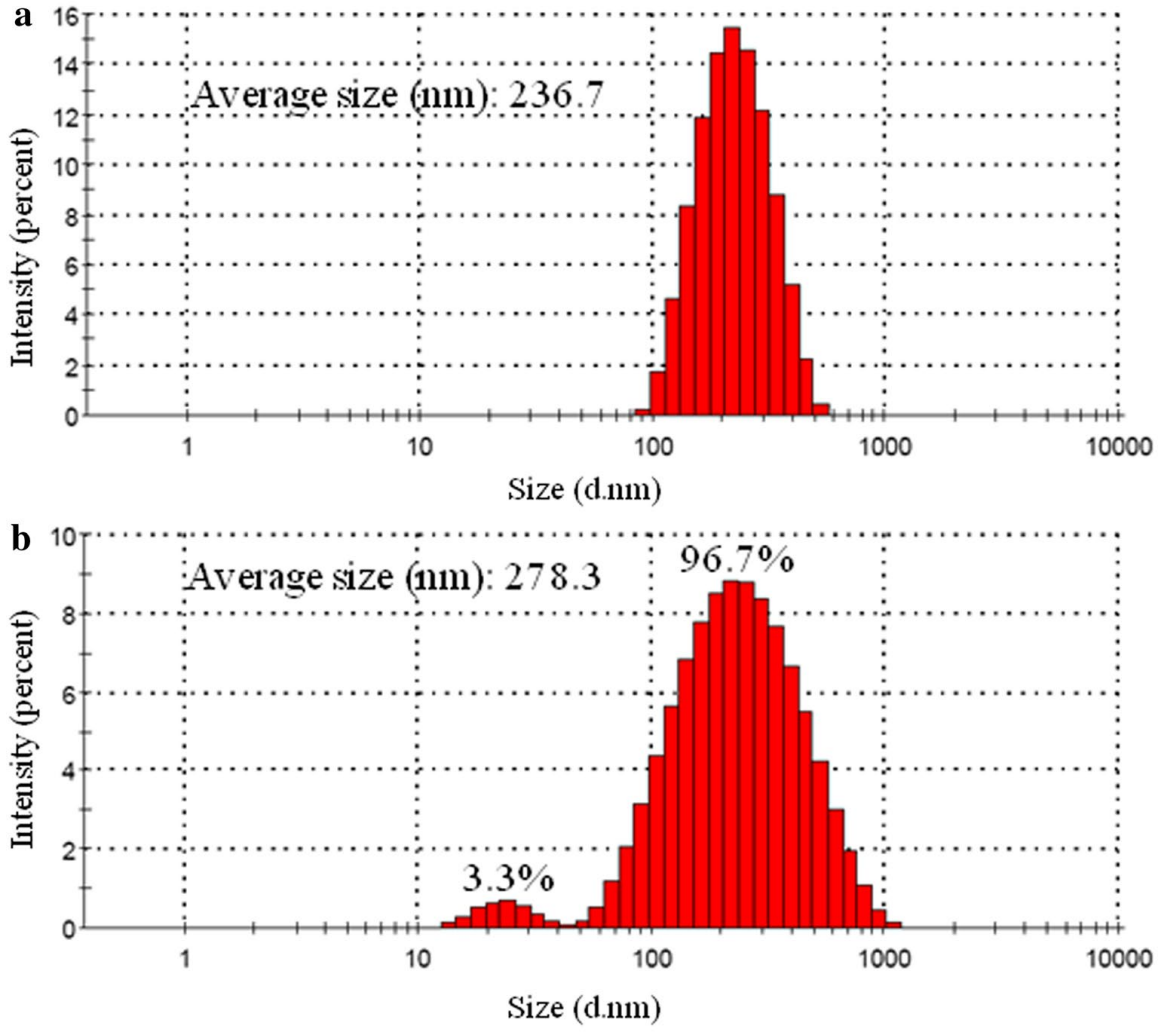
Size distributions of Fe_3_O_4_@NH_2_ FPs and Fe_3_O_4_@*β*_2_-AR FPs with dynamic light scattering. **a** Fe_3_O_4_@NH_2_ FPs. **b** Fe_3_O_4_@*β*_2_-AR FPs

The sizes of control Fe_3_O_4_@NH_2_ FPs and Fe_3_O_4_@*β*_2_-AR FPs were analyzed by DLS with intensity-weighted size distributions (Fig. 3). Both the two types of particles displayed a relatively uniform size distribution, with average sizes of 236.7 nm and 278.3 nm, respectively.

**Fig. 4:**
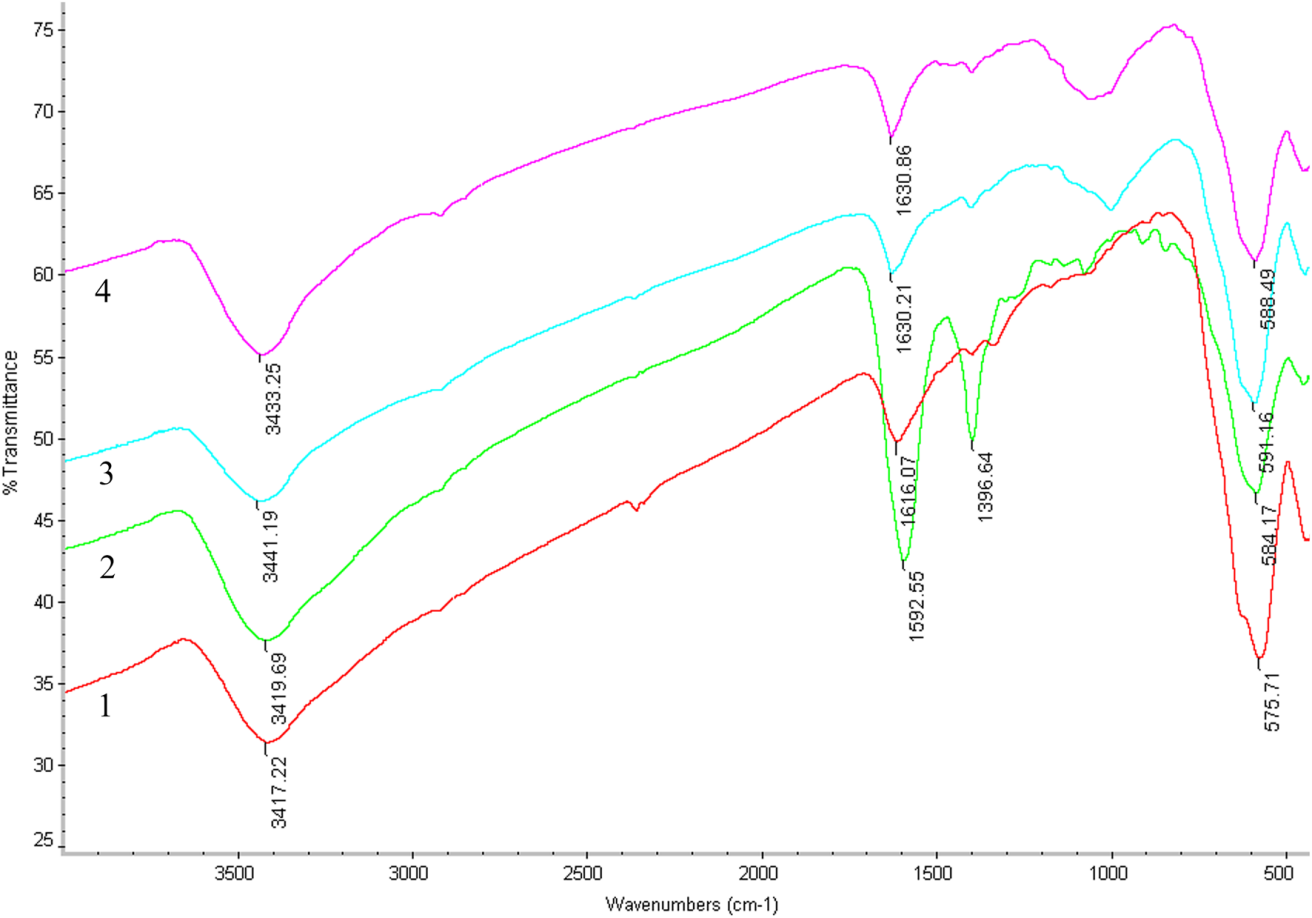
FT-IR spectra of FPs. (1) Fe_3_O_4_ FPs. (2) Sodium citrate solution-modified Fe_3_O_4_ FPs. (3) APTES-modified Fe_3_O_4_ FPs. (4) Fe_3_O_4_@*β*_2_-AR FPs

To determine whether the immobilization of *β*_2_-AR on Fe_3_O_4_ FPs covalent bonds, we analyzed the Fe_3_O_4_@*β*_2_-AR FPs by Fourier transform infrared spectroscopy (FT-IR) after complete rinse of the particles. Figure 4 depicted the representative spectra of Fe_3_O_4_, sodium citrate solution-modified Fe_3_O_4_, APTES-modified Fe_3_O_4_ and Fe_3_O_4_@*β*_2_-AR FPs. The FT-IR adsorption band at 575 cm^−1^ corresponded to Fe–O bond vibrations in iron oxide. The strong absorption band at 3417 cm^−1^ was produced by O–H bond stretching on the Fe_3_O_4_ surface. The absorption peak at 1396 cm^−1^ was the typical absorption peak of carboxylate, providing an evidence for the presence of –COOH on the Fe_3_O_4_ surface. Taking together, the citrate modification of Fe_3_O_4_ was occurred through chemical reaction rather than physical interactions. The spectrum of Fe_3_O_4_-APTES FPs displayed two broad bands at 3441 cm^−1^ and 1630 cm^−1^ due to N–H stretching and NH_2_ bending modes of free NH_2_ group. The strong absorbance observed at 3441 cm^−1^ was attributed to hydrogen bond formation. These results demonstrated that the immobilization of *β*_2_-AR was accomplished by covalent reaction rather than physical absorption.

### Bioactivity of immobilized *β*_2_-AR

The bioactivity of Fe_3_O_4_@*β*_2_-AR FPs was evaluated using salbutamol as a probe. A denaturing buffer solution (10 mM glycine–HCl, pH 2.0) was used to release the bound drug from the particles. The amounts of salbutamol adsorbed on the control and Fe_3_O_4_@*β*_2_-AR NPs surfaces were determined to be 3 ± 1 and 52 ± 2 ng, respectively. The adsorption of metoprolol and prazosin (selective antagonists of *β*_1_-AR and *α*_1_-AR, respectively) on Fe_3_O_4_@*β*_2_-AR FPs was examined under the same conditions. The amounts of the two antagonists on the Fe_3_O_4_@*β*_2_-AR were 2 ± 1 and 3 ± 1 ng. On the basis of the pharmacological properties of the drugs, we concluded that the Fe_3_O_4_@*β*_2_-AR FPs have the bioactivity of recognizing specific ligands of the immobilized receptor. The stability of the immobilized *β*_2_-AR was investigated by measuring the amount of adsorbed salbutamol over 2 weeks. The relative standard deviation (RSD) of the content of the absorbed salbutamol was 2.1% (Table 1), indicating that immobilized *β*_2_-AR was stable for at least 2 weeks.

**Table 1:** Stability investigation of the immobilized *β*_2_-AR MFPs

Times (day)	Salbutamol binding contents (ng)	Relative standard deviation (RSD)
1	58	2.1%
3	56	
7	57	
10	54	
14	53	

The binding contents of salbutamol were determined by denaturing the receptor using 10 mM glycine–HCl (pH 2.0) and releasing the drug into solution

### Competitive displacement of ligands bound on immobilized *β*_2_-AR

**Fig. 5:**
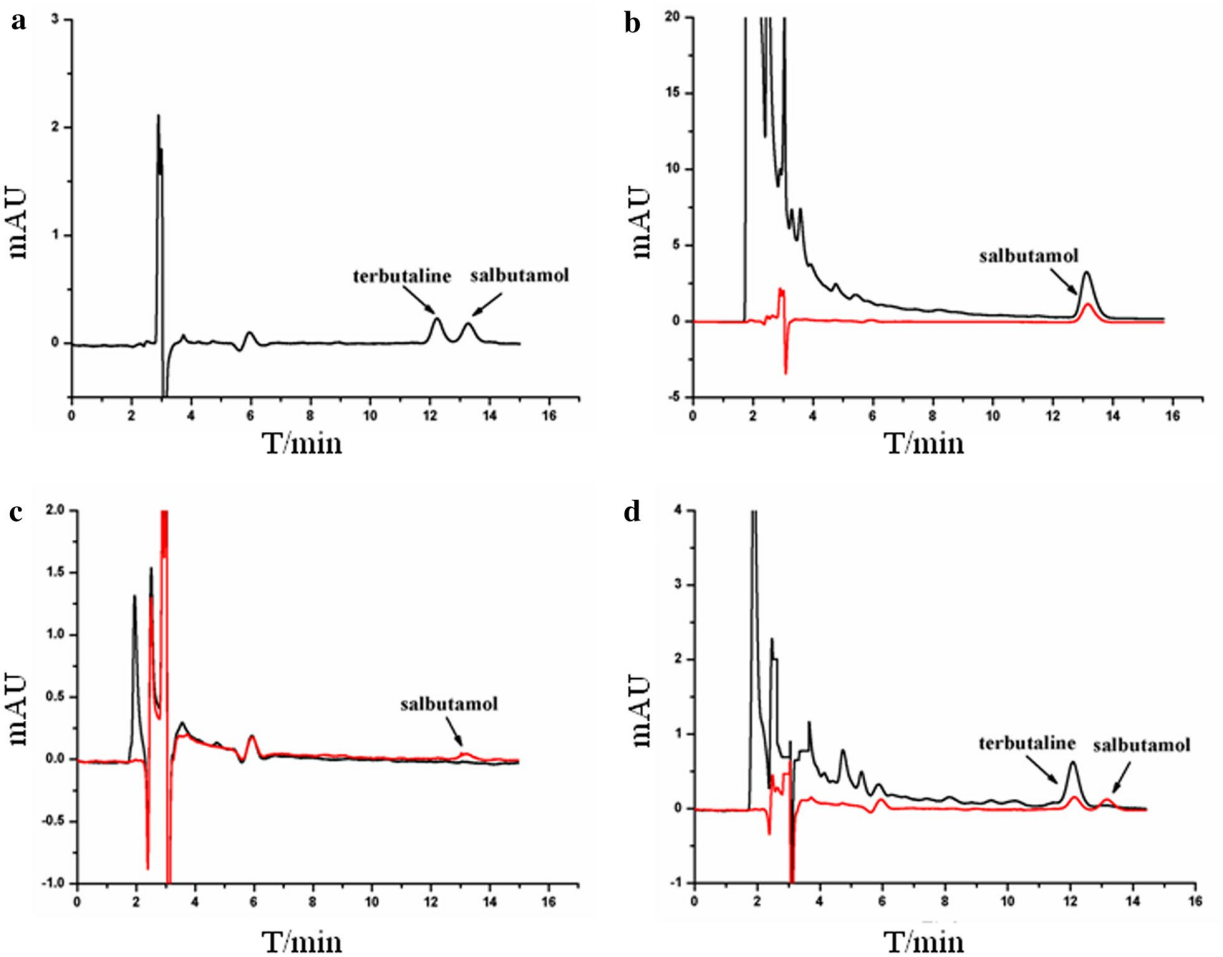
Chromatograms of terbutaline and salbutamol in the collected supernatant from Fe_3_O_4_@NH_2_ FPs (black curve) and Fe_3_O_4_@*β*_2_-AR FPs (red curve) of the competitive displacement. **a** Terbutaline and salbutamol standards. **b** Loading salbutamol to Fe_3_O_4_@*β*_2_-AR FPs. **c** Washing unbound salbutamol by ammonium acetate. **d** Displacing the bound salbutamol from Fe_3_O_4_@*β*_2_-AR FPs by terbutaline

Competitive experiments were performed to prevent false positives which were generated by non-specific binding. Salbutamol was used as a ligand while terbutaline served as a competitive agent. The competitive displacement procedure consisted of three steps: loading, washing, and displacing. In the loading step, we added 200 μL salbutamol (10 μg/mL) to the suspension of Fe_3_O_4_@*β*_2_-AR FPs. Incubating the result suspension for 30 min, we collected the Fe_3_O_4_@*β*_2_-AR FPs with bound salbutamol by a magnet while discarded the free drug in the supernatant. In the second step, we rinsed the Fe_3_O_4_@*β*_2_-AR FPs by ammonium acetate (10 mM, pH 7.4) to remove non-specific binders on the immobilized receptor. In the final step, we incubated the suspension of the clean particles with 2.0 μg/mL terbutaline for 30 min. This aimed to release the bound ligand from the receptor by competitive displacement. Figure 5a showed the chromatograms of salbutamol and terbutaline reference standards. Under the desired conditions, the two drugs were totally separated without any inference. This condition was subsequently utilized to content of the two drugs during the displacement procedure. Figure 5b displayed the chromatograms of salbutamol in the solution post-incubation with control and Fe_3_O_4_@*β*_2_-AR FPs. Compared with the original solution, a clear loss of salbutamol was observed in the supernatant after the incubation with Fe_3_O_4_@*β*_2_-AR FPs. This loss raised from the specific binding of potential ligand onto the immobilized receptor. Figure 5c exhibited the chromatograms of salbutamol in the solution that was utilized to rinse control and Fe_3_O_4_@*β*_2_-AR FPs. The control particles gave no peaks on the chromatograms, while the Fe_3_O_4_@*β*_2_-AR FPs resulted in a weak peak after the same rinsing treatment to control particles. The presence of this peak is expected because there will be a continuous ligand-dissociation/ligand-association from/with the ligand–protein complex at equilibrium [17]. As anticipated, this weak peak disappeared when we rinsed the particles for five times. Considering the speed of the whole methodology, we intended to use two times rinsing for further experiment since the weak peak had little influence on the determination of salbutamol in displacement procedure and was easy to be subtracted. Figure 5d depicted the chromatograms of salbutamol after incubating the Fe_3_O_4_@*β*_2_-AR FPs with terbutaline (competitive agent) solution. Two intensive peaks were observed at 12.2 min and 13.1 min in the chromatogram. Using the reference standards, we identified the two peaks as salbutamol and terbutaline. This result indicated that terbutaline has the capacity to competitively displace the bound salbutamol on the particles. The method is feasible to be utilized for screening potential ligand and simultaneously probing the exact binding sites of the ligand on the receptor.

### Screening bioactive compounds of *β*_2_-AR from SAD

**Fig. 6:**
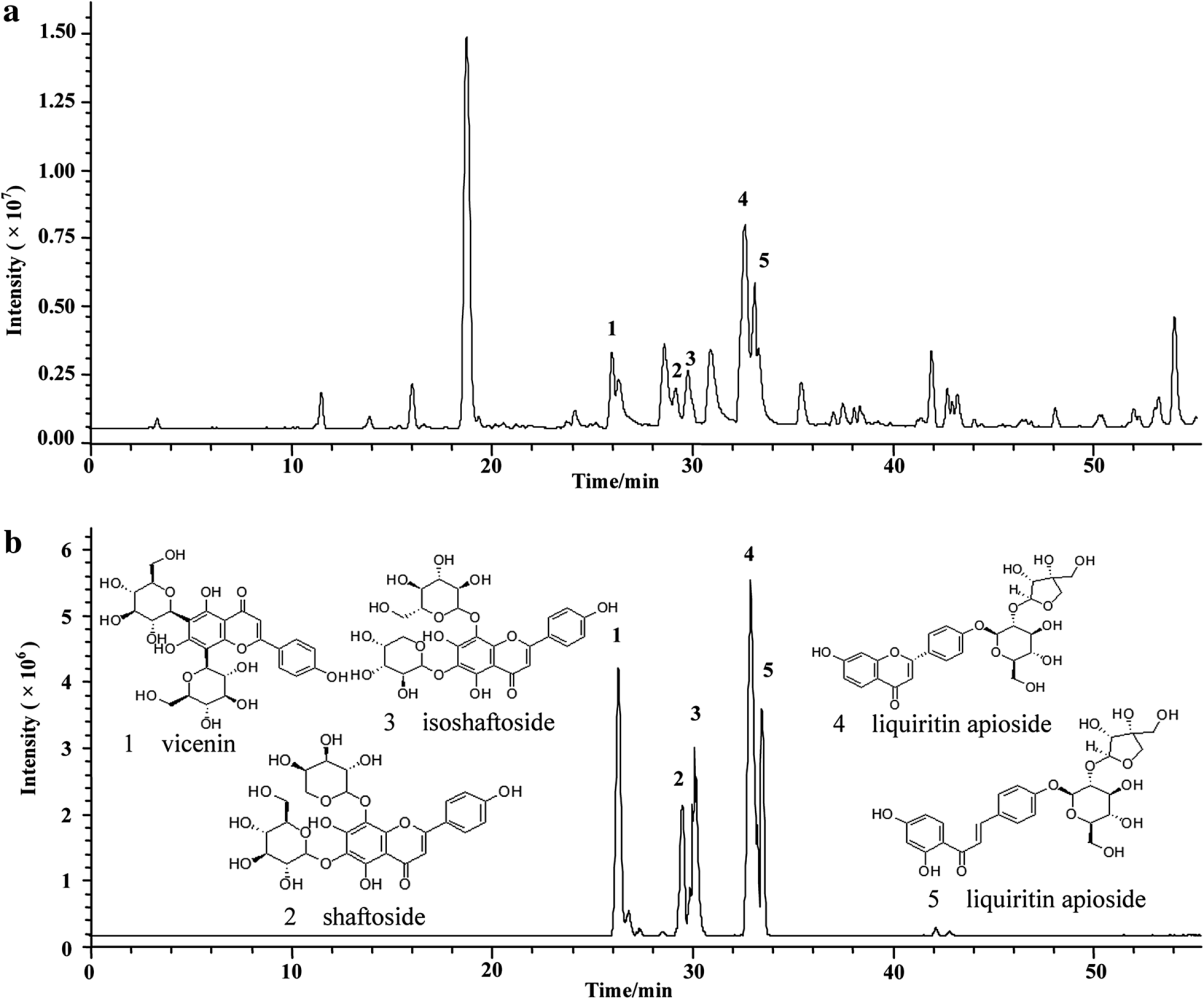
Total ion current chromatograms of SAD extract and the bioactive compounds screened from the prescription. **a** SAD extract. **b** Bioactive compounds screened from SAD

**Fig. 7:**
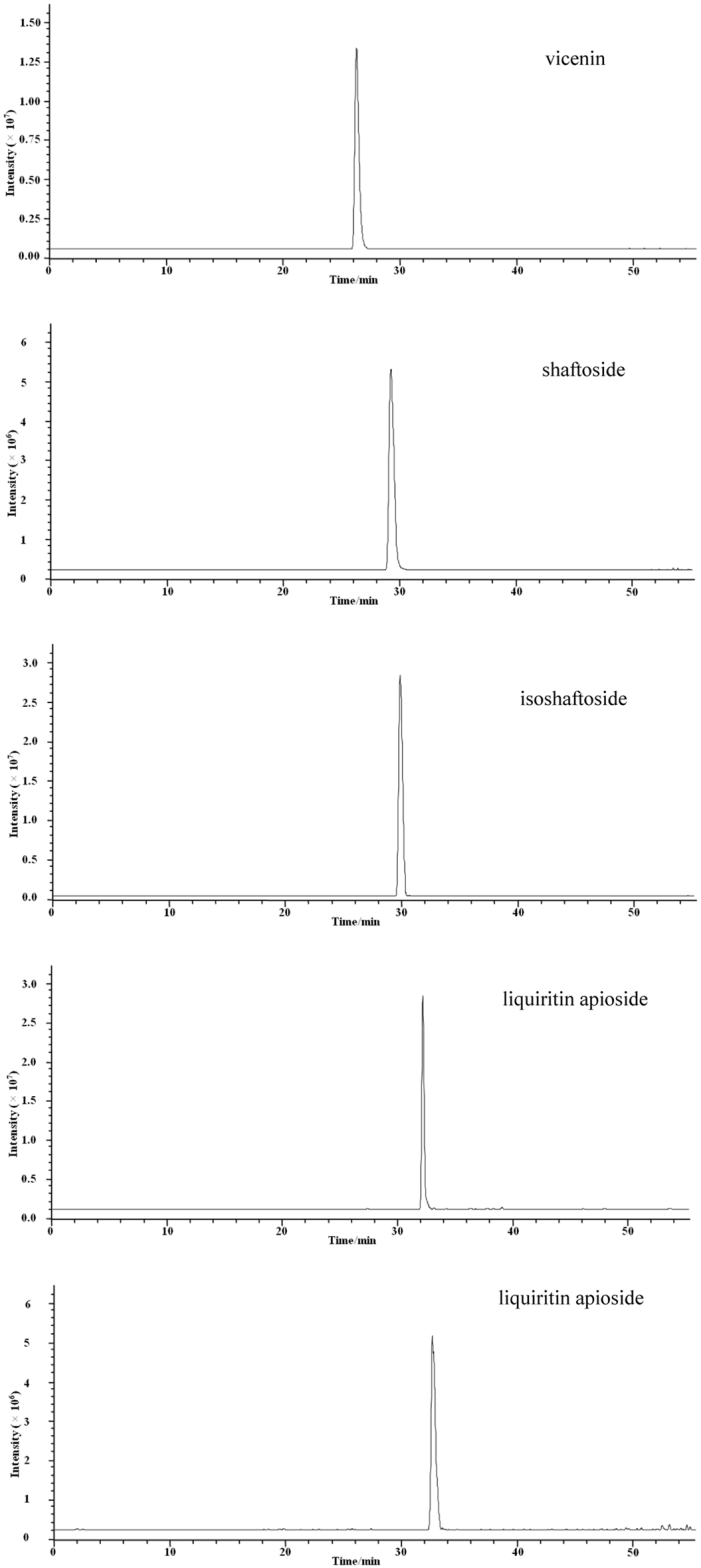
Total ion current chromatograms of the reference standards of vicenin, shaftoside, isoshaftoside, liquiritin apioside and isoliquiritin apioside

The total ion current chromatogram of the SAD extract and the eluted bioactive compounds specifically bound to *β*_2_-AR were illustrated in Fig. 6a, b. Compared with the chromatogram of SAD extract, we observed five intensive peaks after treating the Fe_3_O_4_@*β*_2_-AR FPs with SAD extract. No other peaks in SAD extract were detected in the same sample. These results demonstrated the immobilized receptor has the specificity to capture its ligands from complex matrices. The five peaks were identified by MS/MS analysis and the comparison with mass spectrometric behaviors of reference standards (Fig. 7). The peaks at 26.4 min showed an [M−H]^−^ ion with *m/z* 593.0, which produced daughter ions at *m/z* 503.1, 473.2 and 353.8. This peak was identified as vicenin due to their identical mass patterns. The peaks at 29.2 min and 29.8 min gave same father ion of *m/z* 563.0 [M−H]^−^. This ion generated daughter ions of *m/z* 503.1, 473.2, 443.5, 383.5 and 353.6. Compared with the retention time and mass behavior of reference standards, we attributed this peak to shaftoside and isoshaftoside. The peaks at 32.9 min and 33.4 min generated the same quasi-molecular ion [M−H]^−^ of *m/z* 549.0, which gave main fragment ions of *m/z* 417.0, 297.0 and 255.0. Inspired by previous report and the mass patterns of reference standards, we identified the two peaks as liquiritin apioside and isoliquiritin apioside [34, 35]. In addition, we observed several puny peaks in Fig. 6b. At present, we failed to identify these peaks ascribed to lack of corresponding reference standards and their low intensity which was far away from the requirement of MS/MS analysis.

**Fig. 8:**
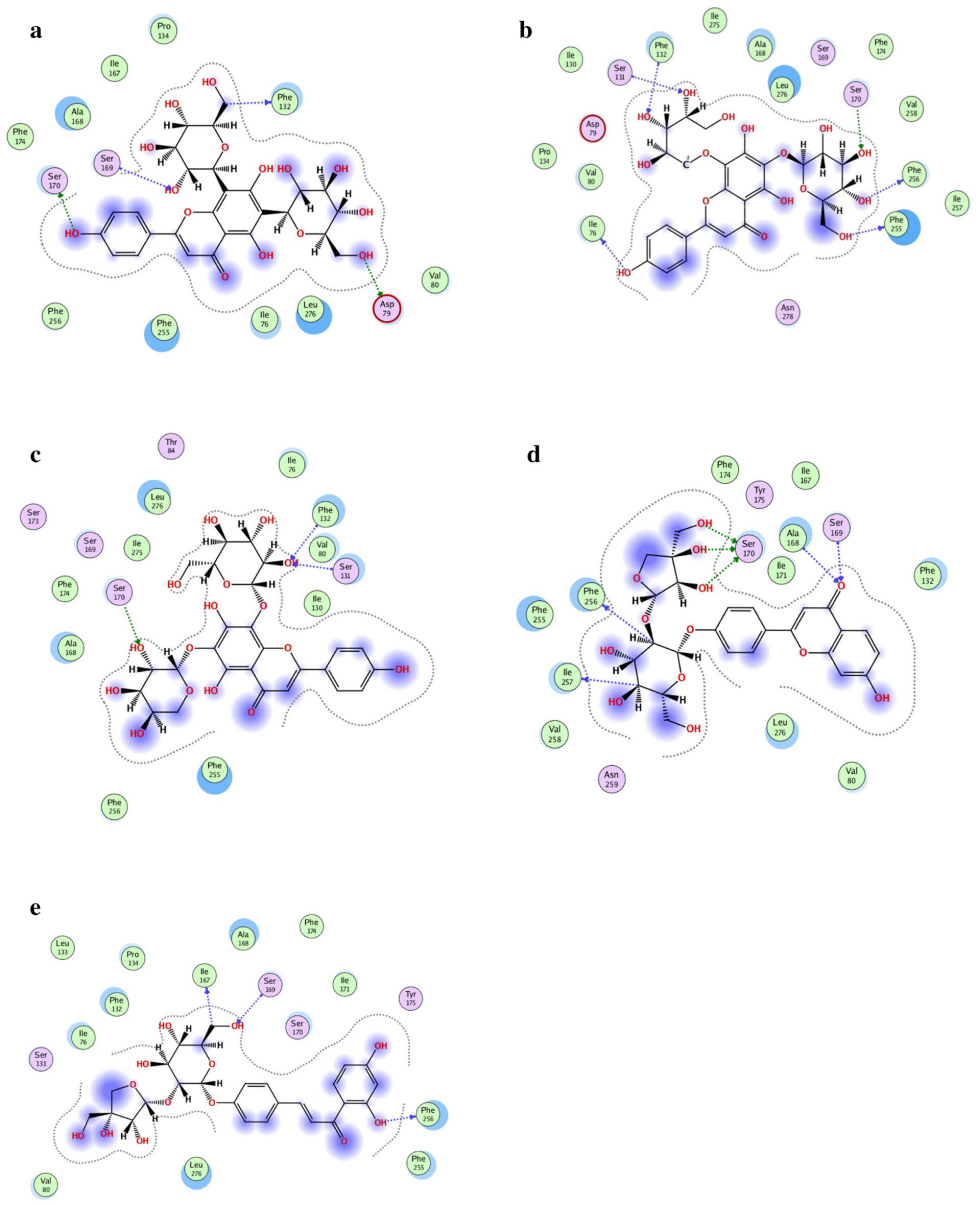
Two-dimensional overview of docking complexations of ligand/*β*_2_-AR. **a** Vicenin, **b** shaftoside, **c** isoshaftoside, **d** liquiritin apioside, **e** isoliquiritin apioside

The interaction between the five compounds and *β*_2_-AR was investigated using molecular docking. As displayed in Fig. 8, serine (Ser) ^169^, Ser^170^ and phenylalanine (Phe)^256^ were found to be the main binding sites of vicenin, shaftoside, isoshaftoside, liquiritin apioside and isoliquiritin apioside to *β*_2_-AR. The driving force of this interaction was the formation of hydrogen bonds between the receptor and the drug.

## Discussion

**Fig. 9:**
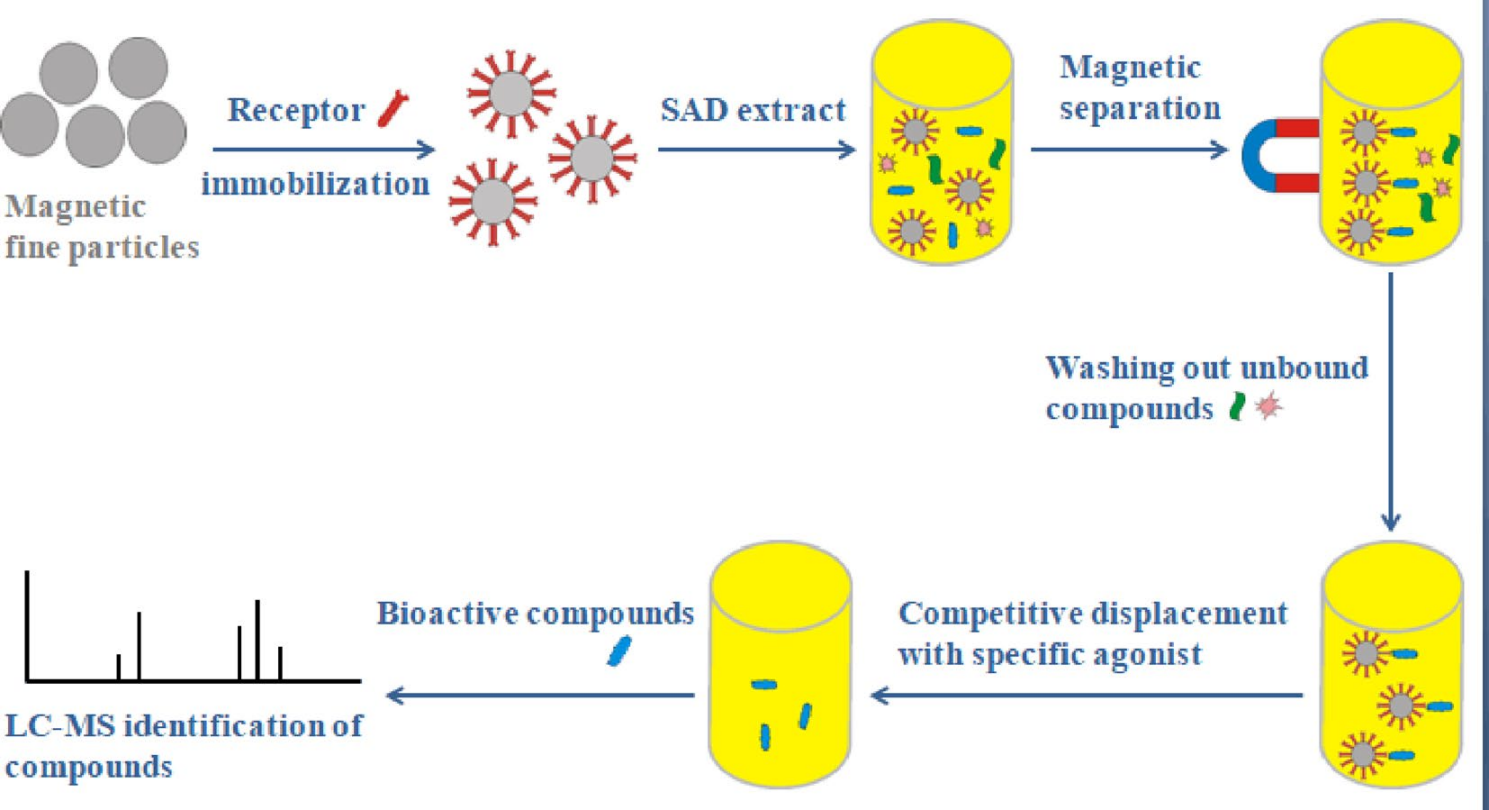
Schematic overview of Fe_3_O_4_@*β*_2_-AR FPs-based ligand screening method. The Fe_3_O_4_@*β*_2_-AR FPs were synthesized by immobilizing the receptor onto Fe_3_O_4_@NH_2_ using N,N′-carbonyldiimidazole as a linking reagent. The bioactive compounds bound to *β*_2_-AR were achieved by incubating the particles with desired samples and were isolated by a magnet. The bound compounds were released into solution by competitive displacement after the particles were totally rinsed. The compounds released from the particles were analyzed by LC–MS/MS to pursue their structures

This was aimed to develop a rapid methodology that enables us to screen the ligands of GPCRs. Development of such methodologies is challenging due to the instability of the receptors when they were removed from their native environment. As illustrated in the scheme of Fig. 9, His-tagged *β*_2_-AR was first immobilized onto Fe_3_O_4_@NH_2_ FPs to achieve an affinity selection supporter. Owning to the large surface area and high biocompatibility of the particles, this immobilization improved the stability of *β*_2_-AR. The Fe_3_O_4_@*β*_2_-AR FPs were used to select bioactive compounds binding to the receptor in a complex prescription. The bound compounds were released from the receptor by competitive displacement using a specific agonist. Finally, HPLC–MS/MS was used to identify the compounds that were released from the Fe_3_O_4_@*β*_2_-AR FPs. Such procedure is attractive since the recognition and isolation of the target compounds are achieved at the same time.

Morphological characterization of the immobilized *β*_2_-AR demonstrated that both the types of particles displayed a relatively uniform size distribution. Compared with Fe_3_O_4_@NH_2_ FPs, the Fe_3_O_4_@*β*_2_-AR FPs produced a size growth of 41.6 nm. We attributed this growth to the immobilization of *β*_2_-AR based on the size of the receptor [36]. The hydroxyl content of the Fe_3_O_4_ FPs was determined to be 384 ± 6.94 μmol/g by acid–base titration. After amino group modification, the hydroxyl content decreased to 43.18 ± 3.21 μmol/g due to reversible Schiff base formation, confirming the covalent modification of Fe_3_O_4_ FPs by APTES. This change became more significant when APTES-modified FPs were treated with *β*_2_-AR, providing a proof of chemical immobilization of the receptor on the particle surface. The molecular weight of the purified protein was determined to be 66.5 kDa by SDS-PAGE. According to this result, we identified the purified protein to be *β*_2_-AR. The quantity of immobilized *β*_2_-AR was determined by the bicinchoninic acid assay. A calibration curve with a regression equation of y = (0.825 ± 0.025)x − (0.0127 ± 0.002) and a correlation coefficient of 0.996 was plotted using bovine serum albumin as the reference standard. Using this curve, the amount of *β*_2_-AR immobilized on the particle surface was determined to be 8.439 ± 0.784 nmol/g. Compared with the immobilization using silica gel as a supporter [37], we declared that the current method was beneficial to achieve a uniform, high density and stable immobilization of the receptor.

Affinity magnetic materials are primarily limited by false positive and false negative results. False positives are caused by nonspecific ligand adsorption, while false negatives are caused by excessive washing steps or manipulation. Considering the mentioned competitive displacement results, we concluded that flushing ligand- Fe_3_O_4_@*β*_2_-AR FPs two times with ammonium acetate was optimal during the washing step. This buffer was compatible with the receptor, and was effective to remove nonspecifically bound moieties and remain high speed without false positive and false negative results.

As a common prescription, SAD is mainly utilized for relieving cough and asthma symptoms in practice [38]. Pharmacological investigation showed that the treatment of asthma with SAD mainly involves the vasodilatations of bronchial vascular smooth muscle, where *β*_2_-AR is the main drug target [39, 40]. These studies indicated that the five compounds were the main bioactive compounds for asthma treatment through mediating *β*_2_-AR signal pathway. Molecular docking results aligned with a previous report states that three sites contribute to the binding of an agonist to *β*_2_-AR. The sites included an aspartate residue on the third domain, two serine residues on the fifth domain and two phenylalanine residues [41]. The agreement of our results with the report confirms the feasibility of Fe_3_O_4_@*β*_2_-AR FPs in screening bioactive compounds of the receptor from complex matrices including traditional Chinese medicine.

## Conclusions

In this work, we synthesized Fe_3_O_4_@*β*_2_-AR FPs to fish out bioactive compounds of the receptor from SAD. Bioactive compounds in SAD that targeted *β*_2_-AR were identified as vicenin, shaftoside, isoshaftoside, liquiritin apioside and isoliquiritin apioside by LC–MS/MS. The compound–receptor interactions occurred at Ser^169^, Ser^170^, and Phe^256^. These results indicated that the affinity magnetic particles have the ability to recognize and separate the target compounds plus the probe of their exact binding site on the receptor. This is probably constitute an effective and rapid method for separating and identifying ligands of GPCRs from complex system such as traditional Chinese medicine.

## Additional file

**Additional file 1.** Minimum standards of reporting checklist.

## Abbreviations

APTES: aminopropyltriethoxysilane
*β*_2_-AR: beta 2-adrenoceptor
DLS: dynamic light scattering
Fe_3_O_4_@NH_2_ FPs: Fe_3_O_4_ fine particles coupled with NH_2_
Fe_3_O_4_@*β*_2_-AR FPs: Fe_3_O_4_ fine particles coupled with the beta 2-adrenoceptor
GPCRs: G protein-coupled receptors
MFPs: magnetic fine particles
OD: optical density
SAD: San-ao decoction
FT-IR: Fourier transform infrared
